# Influence of urbanisation on phytodiversity and some soil properties in riverine wetlands of Bamenda municipality, Cameroon

**DOI:** 10.1038/s41598-022-23278-7

**Published:** 2022-11-17

**Authors:** Godswill A. Asongwe, Irene B. Bame, Lawrence M. Ndam, Ayuk E. Orock, Valantine A. Tellen, Kamah P. Bumtu, Aaron S. Tening

**Affiliations:** 1 Department of Environmental Science, Faculty of Science, University of Buea, P.O. Box-63, Buea, Cameroon; 2 Institute of Agricultural Research for Development (IRAD), P.O. Box 51, Bambui, Bamenda, Cameroon; 3 Deparment of Agronomic and Applied Molecular Sciences, Faculty of Agriculture and Veterinary Medicine, University of Buea, P.O. Box 63, Buea, Cameroon

**Keywords:** Ecology, Environmental sciences

## Abstract

In urban areas, human activities result in the discharge of a variety of chemical substances into the environment. This affects soil quality, plant species diversity and human security. To suggest appropriate management strategies that ensure soil quality and human security amid urbanization, this study assessed the relationship between macrophyte diversity and some soil characteristics of wetlands that are adjacent to urban, peri-urban and rural areas in Bamenda Municipality, Cameroon. Plant communities were sampled for species composition and relative abundance, using the Braun-banquet method. Species richness was evaluated using Simpson’s diversity index. Twenty-one soil samples (0–25 cm depth) were randomly collected within the wetlands and analyzed for their physicochemical characteristics using standard methods. The hierarchical cluster analysis (HCA) was used to group the wetlands under managing units. The dominant species ranked in order of abundance in the rural wetland were *Raphia farinifera* > *Ludwigia hexandra* > *Coix* spp. > *Leersia hexandra* > *Ehchinochloa paramidelis.* The Peri-urban wetland dominant species stood at *Commelina bengalensis* > *Leersia hexandra* > *Cyperus distance* > *Ehchinochloa pyramidalis.* In the Urban segment, *Pennisetum purpureum* > *Echinochloa pyramidalis* > *Tithonia diversifolia* > *Leersia hexandr*a were the abundant species. The Simpson index of diversity was 0.94 for the urban and 0.96 for the peri-urban and rural sites respectively. The soils were slightly acidic with pH KCl ranging from 4.87 to 5.41. From the coefficient of variability classes, Sand, pH-H_2_O, pH-KCl, and Na consistently varied slightly across the three sites. Two significant clusters (management units) representing a combination of urban, and peri-urban/rural were formed from the hierarchical dendrograms. The Mann–Whitney *U* test revealed a significant (*P* < 0.05) lower exchange acidity in the rural than the urban sites indicating contamination of the urban site, reducing its macrophyte diversity. Intensification and extension of urbanisation are gradually reducing the macrophyte diversity and also contaminating the soils of the wetlands of the Bamenda municipality in Cameroon, warranting monitoring. The chemical composition of soils in the urban cluster needs early remediation by encouraging the planting and monitoring of certain plants that can already take up the elements.

## Introduction

Human activities such as agriculture and urbanisation, represent an existential problem between nature and society in relation to phytodiversity, soil quality, landscape alteration and global warming^1–4^. Phyto-diversity is one of the indicators often used to measure the well-being of ecological systems^5^. In tropical regions, the structure and diversity of vegetation are determined by several biotic and/or abiotic factors, which act on different spatial and temporal scales^6^

Topography and soil type are among the major abiotic factors that play a major role in the heterogeneity of habitats and species, thus contributing to the physiognomic differentiation of the vegetation^7,8^.

In urban areas, human activities result in the discharge of a variety of chemical substances into the environment that alters the quality of the soil. This ultimately results in changes in the structure of plant communities, their species diversity and human security.

Plants and soils are intricately related to an extent that changes in any of them would alter the other significantly^9^. This could be partly associated with the fact that the fertility of soils is strongly influenced by the types of plants that grow on them. The types of plants influence the development of soil profiles^9^.

Wetlands in urban and peri-urban areas can provide a range of important ecosystem services and benefits to people. However, in many countries, just as in Cameroon, as a result of spreading urbanization, wetlands are increasingly becoming degraded. The deposition of wastes in these zones also defies the Cameroon law on the Environment, Section III article 31(1) which forbids the deposition of waste into natural drains^10^. ^11^ and ^12^ observed that industrial and domestic wastes deposited in water add large amounts of inorganic substances ranging from trace metals to acids and alkalis that are potential pollutants in aquatic systems. The deposition of wastes into these systems, therefore, portends danger. Macrophytes are important components of wetlands where they grow vigorously in enriched areas with a species variety that is strongly related to the chemical constituents of the environment. They have high remediation potentials for macro and micronutrients because of their general fast growth and high biomass production in contaminated areas. In this regard, emergent and floating species especially those commonly used in the treatment of wastewater^13^ are good indicators of environmental degradation in wetlands.

This study assesses the relationship between macrophyte diversity and some soil characteristics (physical and chemical) of wetlands in Bamenda Municipality, Cameroon at different stages of urbanisation.

## Materials and methods

### Description of the study area

**Figure 1:**
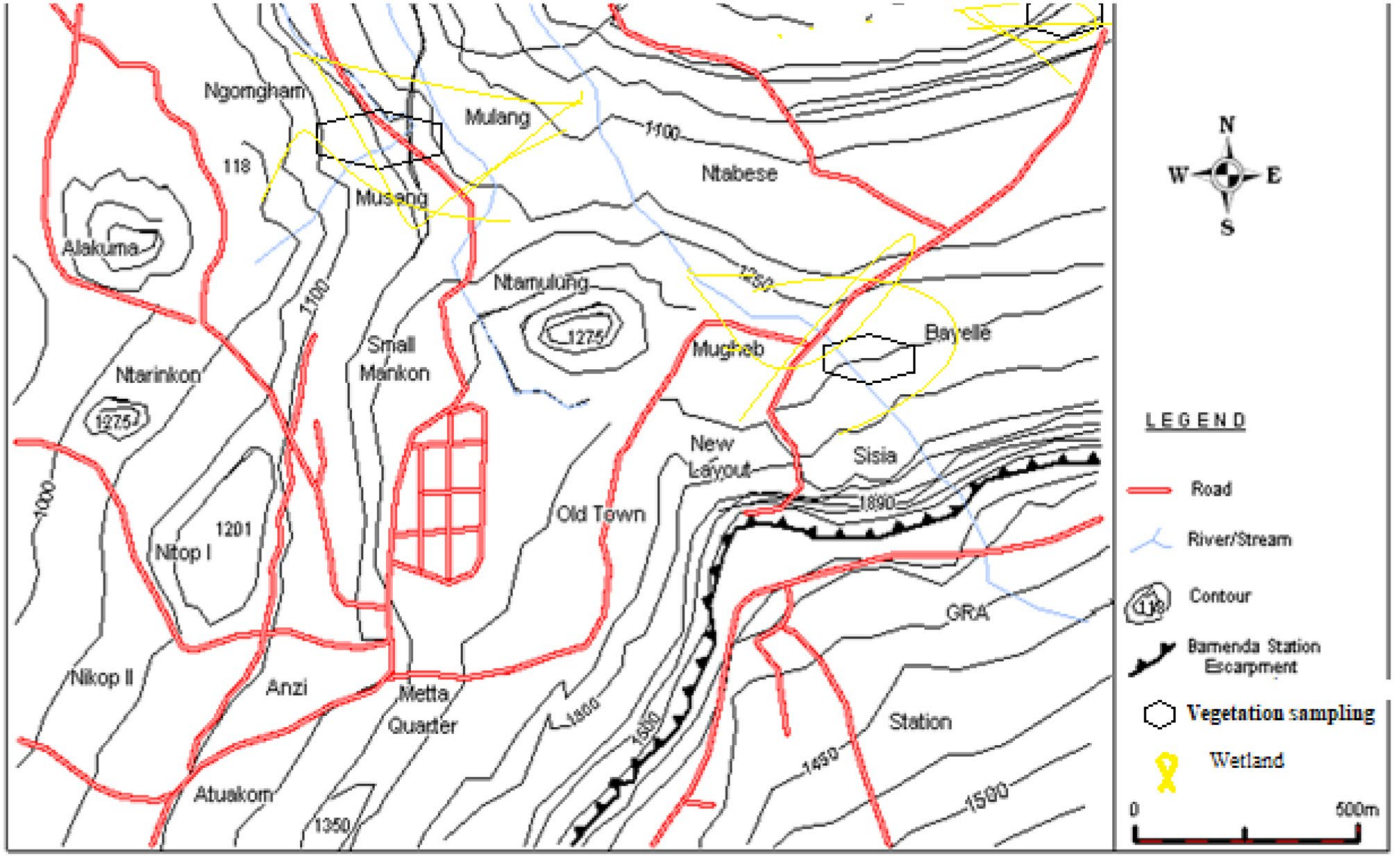
Relief Map of Bamenda showing the Bamenda escarpment, topography and the location for quadrat sites.

The study covers urban, peri-urban and rural wetlands in the Bamenda Municipality of the North West Region of Cameroon that have evolved concomitantly with different stages of urbanization (Fig. 1). In this study, urbanisation is considered a loose term that is aimed at giving a geographical expression to the variation in the economic, social and cultural practices in the milieu. The central town with many economic activities is termed the urban, the fringe area with sprawls is termed peri-urban while the rural has typical peasant activities and make-shift structures. From the variation of human activities in the three sub-zones, a variety of chemical substances are discharged into drains, playing a substantial role in soil quality, and therefore plant macrophyte diversity. The Plants studied were ubiquitous in the area and verification of their IUCN conservation status in the red data book of plants of Cameroon confirmed their abundance^14^. Information on protected sites in Cameroon does not place the study area under conservation status. In line with that, permits are not required to undertake academic and research studies as well as do a responsible collection of plants in the study area. The urbanization rate of Bamenda is 42%, and the population grew from 48,111 inhabitants in 1976 to 488,883 inhabitants in 2010^15^, with 150–200 inhabitants/km^2^.

The study area is part of the Bamenda escarpment that is located between latitudes 5° 55″ N and 6° 30″ N and longitudes 10° 25″ E and 10° 67″ E. The town shows an altitudinal range of 1200–1700 m and is divided into two parts by escarpments—a low-lying and gently undulating part with altitudes ranging from 1200 to 1400 m, with many flat areas that are usually inundated for most parts of the year, and an elevated part that range from 1400 to 1700 m altitude. Most of the streams take their rise from this elevated part (Fig. 1).

This area experiences two seasons—a rainy season (mid-March to mid-October) and a short dry season (mid-October to mid-March). The thermic and hyperthermic temperature regimes dominate in the area. The mean annual temperature stands at 19.9 °C. January and February are the hottest months with mean monthly temperatures of 29.1 and 29.7 °C, respectively. This area is dominated by the Ustic and Udic moisture regimes with the Udic extending to the south^9^. Annual rainfall ranges from 1300 to 3000 mm^16^. The area has a rich hydrographical network with intense human activities and a dense population along different water courses in the watershed. The area is bounded on the West, North and East by the Cameroon Volcanic Line (made up of basalts, trachytes, rhyolites and numerous salt springs). The geologic history of this area originates from the Precambrian era when there was a vast formation of geosynclinal complexes, which became filled with clay-calcareous, and sandstone sediments^9^. These materials, crossed by intrusions of crystalline rocks, were folded in a generally NE-SW direction and underwent variable metamorphism^9^. The Rocks in the area are thus of igneous (granitic and volcanic) and metamorphic (migmatites) origin^17^, which gives rise to ferralitic soils^18^.

Agriculture is the principal human activity in and around this region^18^. The area equally harbours the commercial center that has factories ranging from soap production, and mechanic workshops to metallurgy, which may be potential sources of pollutants that can influence wetland Geochemistry. *Raffia farinifera* bush, which is largely limited to the wetlands, is an important vegetation type in this area. *R. farinifera* provides raffia wine, a vital economic resource to the inhabitants who are fighting against the cultivation of these wetlands by vegetable farmers.

### Methods of the study

#### Macrophyte diversity study

The plant diversity of the wetlands was evaluated using quadrats in the dry season for accessibility reasons. For each of the three wetlands (the urban, peri-urban and rural areas), three transects were established on which representative quadrats, each measuring 10 m × 10 m, were mapped out in uncultivated areas for the determination of plant species cover-abundance and diversity. It is perceived that the different zones receive different mixtures of chemical substances and thus influence macrophyte diversity differently.

According to a publication by^14^ on the vascular plants of Cameroon and a taxonomic checklist with IUCN assessment, the plants of the area are placed under the Least Concern Category(LC), and therefore not in the risky category. Diversity studies involved the identification of a specific area called “relevé” by progressively increasing test quadrat size and sampling for specific diversity until the smallest area with adequate species representation was reached. The relevé size determined here was 1 m^2^, making a total of 300 sub-quadrats (relevé) in the entire study ie. 100 in each main quadrat). For each site (main quadrat), 10 representative relevés were sampled and all plant species were enumerated. Most plant species in each of them were identified in the field by the Botanist, Dr Ndam Lawrence Monah using visual observation of the morphology of the leaves and flowers, a self-made field guide, the Flora of West Africa and the Flora of Cameroon. 10 unidentified plants were appropriately collected where there were in abundance, placed onto a conventional plant press and dried in the field. Voucher specimens were tagged and transported to the Limbe Botanic Gardens (SCA: Southern Cameroon, the code of the Limbe Botanic Gardens Herbarium) for identification. Mr Elias Ndive, the Taxonomist of the Limbe Botanic Gardens compared unidentified specimens with authentic herbarium specimens and other taxonomic guides and finally identified them. Voucher specimens of the 10 plants were given identification numbers and deposited in the Herbarium of the Limbe Botanic Gardens.

The Braun–Banquet method was used^19^ for the assessment of species cover abundance. Relative abundance and abundance ratings were determined using the Braun–Banquet rating scheme (Table 1).

**Table 1:** Braun-Blanquet rating scheme for vegetation cover-abundance, *Source*^19^.

Class	% range of cover	Mean	Rating
5	75 – 100	87.5	Very numerous
4	50 – 75	62.5	Numerous
3	25 – 50	37.5	Not numerous
2	5 – 25	15.0	Occasional
1	1 – 5	2.5	Sparse
R	< 1	–	–

#### Simpson’s diversity index

Species richness was evaluated using Simpson’s diversity index (D), which takes into account both species richness and the Braun-Blanquet rating scheme for vegetation cover abundance and evenness of abundance among the species present. In essence, D measures the probability that two individuals that are randomly selected from an area will belong to the same species. The formula for calculating D is presented as:

$${\text{D}} = \frac{{\sum {{\text{n}}_{i} \left( {{\text{n}}_{i} - 1} \right)} }}{{{\text{N}}({\text{N}} - 1)}}$$

where n_*i*_ = the total number of each species; N = the total number of individuals of all species.

The value of **D** ranges from 0 to 1. With this index, 0 represents infinite diversity and 1 represents no diversity. That is, the larger the value the lower the diversity.

Alternatively, **Simpson’s Diversity Index, = 1–D,**

**1-D** was used as a measure of diversity because it is more logical and less likely to cause confusion. The scale then gives a score from 0 to 1 with higher scores showing higher diversity (instead of being associated with low scores).

The Simpson index is a dominance index because it gives more weight to common or dominant species. In this case, a few rare species with only a few representatives will not affect the diversity.

#### Soil sampling and analysis

**Figure 2:**
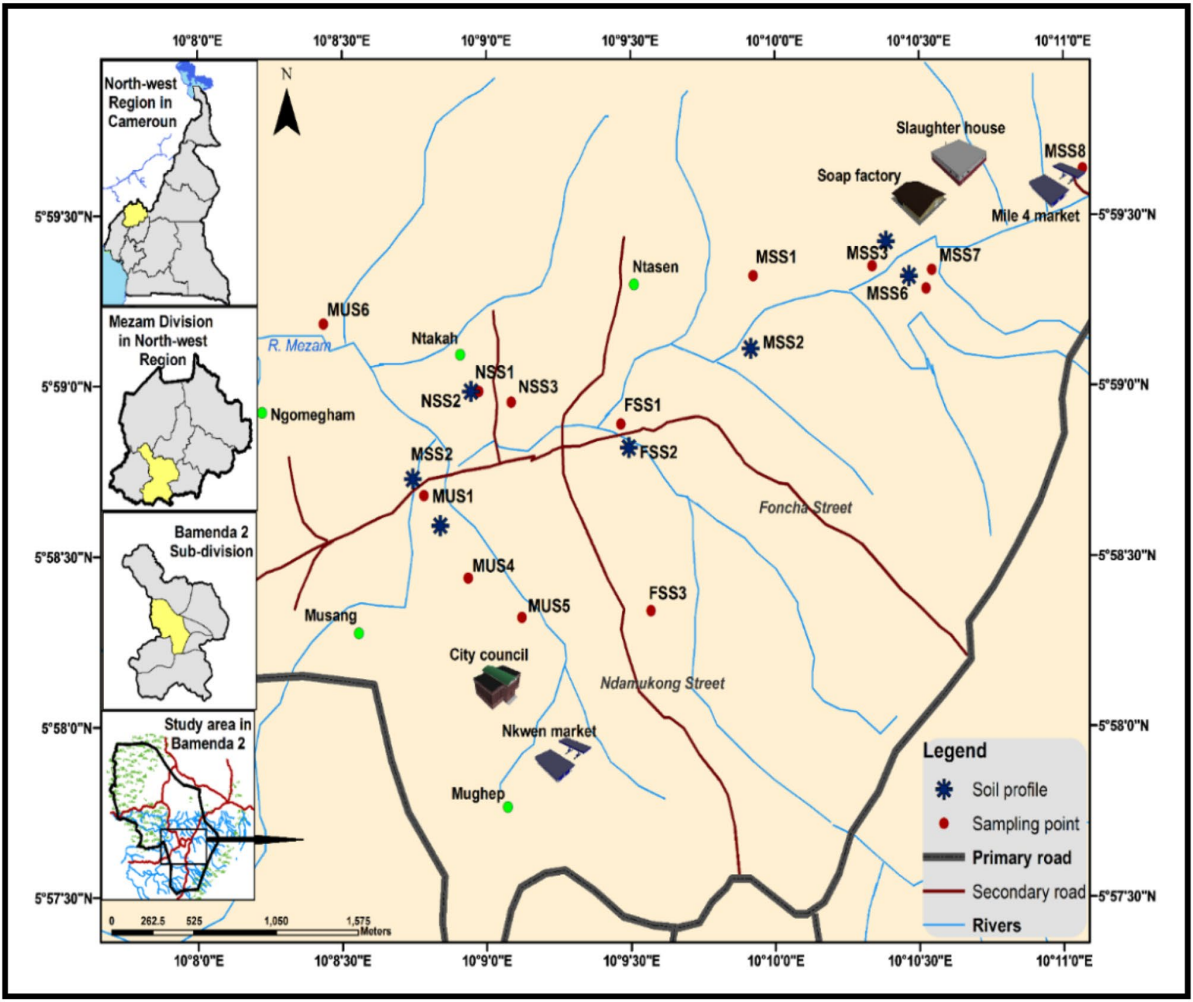
Map of the study area in freshwater wetlands of Bamenda Municipality. Adapted from the 1980 land use map of the Bamenda City Area showing soil sampling points: Source Bamenda City Council.

Soil sampling was done in and around the three quadrats laid in the urban, peri-urban and rural wetlands for macrophytes sampling. Twenty-one (21) composite samples (0–25 cm) were randomly collected (Fig. 2) and taken to the laboratory in black plastic bags. Each composite sample was a collection of 5 dried core soil samples. Due to the observed greater heterogeneity in the urban sector, the sampling density was intensified. The soil samples were air-dried and screened through a 2-mm sieve. They were analyzed in duplicate for their physicochemical properties in the Environmental and Analytical Chemistry Laboratory of the University of Dschang, Cameroon. Particle size distribution, cation exchange capacity (CEC), exchangeable bases, electrical conductivity (EC) and pH were determined by standard procedures^20^. Soil pH was measured both in water and KCl (1:2.5 soil/water mixture) using a glass electrode pH meter. Part of the soil was ball-milled for organic carbon (Walkley–Black method) and total nitrogen (Macro-Kjeldahl method) as largely described by^20^. Available phosphorus (P) was determined by Bray I method. Exchangeable cations were extracted using 1 N ammonium acetate at pH 7. Potassium (K) and sodium (Na) in the extract were determined using a flame photometer and magnesium (Mg) and calcium (Ca) were determined by complexiometric titration. Exchange acidity was extracted with 1 M KCl followed by quantification of Al and H by titration^20^. Effective cation exchange capacity (ECEC) was determined as the sum of bases and exchanged acidity.

Apparent CEC (CEC at pH 7) was determined directly as outlined by^20^. Based on critical values of nutrients established for vegetables, nutrients were declared sufficient or deficient.

### Statistical analysis

The data were subjected to statistical analysis using Microsoft Excel 2007 and SPSS statistical package 20.0. Soil properties were assessed for their variability using the coefficient of variation (CV) and compared with variability classes (Table 2).

$$CV=\frac{Sd}{X}X 100$$

where: Sd = standard deviation; = *X* arithmetic mean of soil properties.

**Table 2:** Grouping coefficient of variation into variability classes.

. CV (%)	Variability grouping (class)
< 15	Slightly variable
15–35	Moderately variable
> 35	Highly variable

The hierarchical cluster analysis (HCA) was used to group the area under managing units. The main goal of the hierarchical agglomerative cluster analysis is to spontaneously classify the data into groups of similarity (clusters). This is done by searching objects in the n-dimensional space that is located in the closest neighborhood and separating a stable cluster from other clusters. The sampling sites were considered objects for classification. Each object was determined by a set of variables (chemical concentrations of the soils in this case).

## Results

### Macrophyte biodiversity study

**Figure 3:**
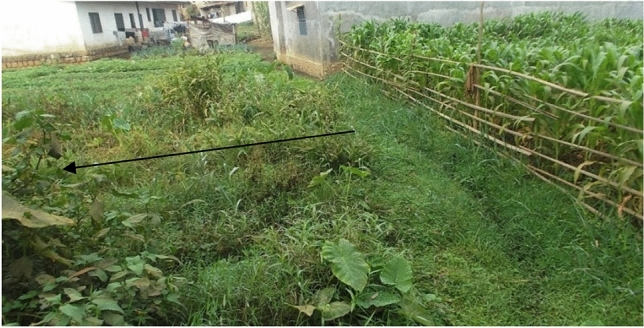
Discoloration of vegetation along a waste channel in the urban wetland of Bamenda Municipality.

Semi-aquatic and marshland plant species in the wetlands were mainly herbaceous and shrubby. Most of the plants showed discoloration in patches throughout the wetland (Fig. 3). Human activities that involved the cutting of raffias and woody species, resulted in open vegetation with shrubs scattered all over the wetlands.

A checklist of plant diversity in agricultural wetlands in the Bamenda Municipality (Table 2) shows that Macrophytes constituted an important part of the wetland.

**Figure 4:**
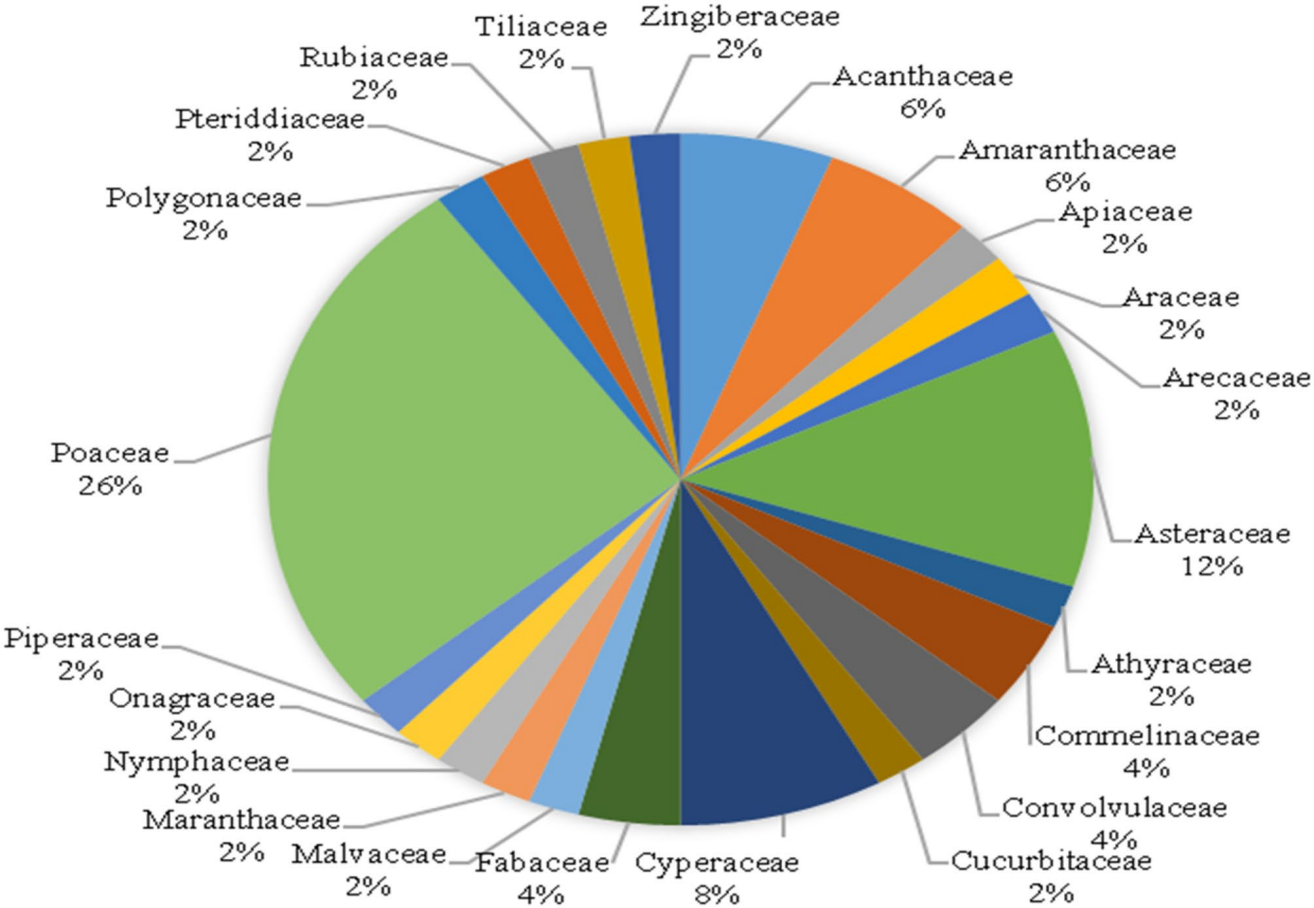
Percent distribution of riverine wetland flora families in Bamenda Municipality.

From the quadrat studies, 50 plants (macrophytes) species distributed in 28 families were identified in the wetlands (Fig. 4). The species observed were mainly emergent herbaceous plants (grasses) with only a few shrubs and trees. The Poaceae (26%) occurred as the most represented family, followed by Asteraceae (12%), Cyperaceae (8%), Acanthaceae (6%), and Amaranthaceae (6%) with other species less represented (Fig. 4). The marshland from visual observation was dominated by *Leersia hexandra* growing in open water and covering large areas.

The rural site at Mbelewa was represented by 39 species. Here, the dominant species ranked in abundance were *Raphia farinifera* > *Ludwigia hexandra* > *Coix spp.* > *Leersia hexandra* > *Ehchinochloa paramidelis.*

The Peri-urban wetland was represented by 40 species. Here, the dominant species were ranked in abundance as *Commelina bengalensis* > *Leersia hexandra* > *Cyperus distance* > *Ehchinochloa pyramidalis.* At the Urban segment, *Pennisetum purpureum* > *Echinochloa pyramidalis* > *Tithonia diversifolia* > *Leersia hexandra* were the abundant species. *Pennisetum purpureum*, *Echinochloa pyramidalis* and *Tithonia diversifolia* lined the main river course.

From the study, the Simpson index of diversity were 0.94 each at urban (Table 3), and peri-urban (Table 4) sites, lower than the 0.96 value recorded at the rural or the control site (Table 5). This indicates that the Simpson’s Diversity index value of 0.94 and 0.96 is so close in the three settings.

**Table 3:** Checklist of macrophytes diversity in the wetlands in Bamenda municipality, Cameroon.

Family	*Species*	Class	Voucher no	Common name	Count (n)	n-1	n(n-1)
Acanthaceae	*Brilantasia nitens Lindau*	Dicot	–	–	3	2	6
	*Decliptera laxata C. B. Clarke*	Dicot	SCA: 1087	–	12	11	132
	*Justicia obliquifolia Standl*	Dicot	–	Blood medicine	2	1	2
	*Nesonia canenscens Var. Smithii*	Dicot	–	–	5	4	20
Amaranthaceae	*Alternanthera sessalis* L	Dicot	–	Sessile joy weed	4	3	12
	*Amaranthus hybridus* L	Dicot	–	–	15	14	210
	*Amaranthuss spinosus* L	Dicot	–	Spiny amaranth	12	11	132
Apiaceae	*Centella asiatica* (L.) *Urb*	Dicot	–	Penny wort	5	4	20
Araceae	*Colocasia esculentus* L	Dicot	–	Cocoyam	3	2	6
Arecaceae	*Raffia fanivera Gaertnb*(L)	Monocot	–	Raffia palm	3	2	6
Asteraceae	*Ageratum cornyzoides.* L	Dicot	–	King grass	12	11	132
	*Tithonia diversifolia Hemsl*	Dicot	–	Sun flower	35	34	1190
	*Vernonia amygdalina Del*	Dicot	–	Bitter leaf	9	8	72
	*Vernonia hymenolepis A. Rich*	Dicot	–	Sweet bitter leaf	4	3	12
Casiapinaceae	*Cassia alata* L	Dicot	–	Leaves like that of guava	2	1	2
Commelinaceae	*Commelina beghalensis* L	Dicot	SCA: 1225	–	43	42	1806
	*Commelina diffusa* (Burn) *F*	Dicot	–	Tropical sidewort	10	9	90
Convolvulaceae	*Ipomoea aquatic Forsk*	Dicot	SCA: 2733	–	9	8	72
	*Ipomea batatas* L	Dicot	–	Sweet potatoes	5	4	20
Curcubitaceae	*Zsheria scarbra* (L.F.) *Sond*	Dicot	–	Curcumber	4	3	12
Cyperaceae	*Cyperus distans*	Monocot	–	–	15	14	210
Dryopteridaceae	*Dryopteris felix-mas*(L) *Schoot*	Dicot	–	Fern plant	2	1	2
Fabaceae	*Centrosoma pubescens* (D.C.) *Benth*	Dicot	SCA: 2526	–	3	2	6
	*Desmodium salicifolium* (Poir.) *DC*	Dicot	–	–	11	10	110
Maranthaceae	*Marantchloa purpurea* (Ridl.) *Milne-Redh*	Dicot	SCA: 1351	–	1	0	0
Mimosaceae	*Mimosa pudica*	Dicot	–	–	2	1	2
Onagraceae	*Ludwigia abysisnica A. Rich*	Dicot	–	Water primerose	12	11	132
Poaceae	*Anzonopus compressus* (Sw.) *P. Beauv*	Monocot	–	Flat leaf carpet grass	7	6	42
	*Cyndon dactylon* (Linn)	Monocot	–	Bermuda grass	16	15	240
	*Digitaria sanguinalis*(L)	Monocot	–	Crab grass	14	13	182
	*Echinochloa crus-pavonis* (Kunth) *Schult*	Monocot	SCA: 6075	–	17	16	272
	*Echinochloa paramidelis* (Lam.) *Hitchc*	Monocot	–	–	13	12	156
	*Eragrostis* spp. *Michx*	Monocot	–	–	10	9	90
	*Imperata cylindica*	Monocot	–	Spear grass	15	14	210
	*Leersia hexandra* Sw	Monocot	–	–	67	66	4422
	*Panicum maximum Jacq. Var*	Monocot	–	Guinea grass	10	9	90
	*Pennisetum purpereum* (Schumach)	Monocot	–	Elephant grass	54	53	2862
	*Saccharum officinarium* (L)	Monocot	–	sugar cane	10	9	90
	*Pteridium equilinum*	Dicot	–	Braken fern	4	3	12
	*Polygonium limbatum Meisn*	Dicot	–	–	12	11	132
Portuleraceae	*Portulaca oleraceae* L	Dicot	–	–	3	2	6
Rubiaceae	*Spermacoce latifolia Aubl*	Dicot	–	–	10	9	90
Total	42				505		13,312

*SCA* Southern Cameroon (code for the Limbe Botanic Gaderns), D = 0.052302, Simpson index = 0.94.

**Table 4:** Simpson index of diversity from vegetation samples at the Peri-urban site (Mile Four) in the Bamenda Municipality.

Family	Species	Class	Voucher no	Common name	count (n)	n-1	n(n-1)
Acanthaceae	*Brilantasia nitens Lindau*	Dicot	–		20	19	380
	*Decliptera laxata C. B. Clarke*	Dicot	SCA: 1087	–	22	21	462
	*Justicia obliquifolia Standl*	Dicot	–	Blood medicine	7	6	42
Amaranthaceae	*Alternanthera sessalis* L	Dicot	–	Sessile joy weed	3	2	6
	*Amaranthus hybridus* L	Dicot	–	–	11	10	110
	*Amaranthuss spinosus* L	Dicot	–	Spiny amaranth	13	12	156
Apiaceae	*Centella asiatica* (L.) *Urb*	Dicot	–	Penny wort	5	4	20
Araceae	*Colocasia esculentus* L	Dicot	–	Cocoyam	8	7	56
Arecaceae	*Raffia fanivera Gaertnb*(L.)	Monocot	–	Raffia palm	19	18	342
Asteraceae	*Ageratum cornyzoides.* L	Dicot	–	King grass	22	21	462
	*Tithonia diversifolia Hemsl*	Dicot	–	Sunflower	21	20	420
	*Vernonia amygdalina Del*	Dicot	–	Bitter leaves	16	15	240
	*Vernonia hymenolepis A. Rich*	Dicot	–	Sweet bitter leaves	14	13	182
	*Spilanthes filicaulis* (Schum. & Thonn.) *C. D. Adams*	Dicot	–	Eye for fowl	18	17	306
Commelinaceae	*Commelina beghalensis* L	Dicot	SCA: 1225	–	119	118	14,042
	*Commelina diffusa* (Burn.) *F*	Dicot	–	Tropical sidewort	16	15	240
Convolvulaceae	*Ipomoea aquatic Forsk*	Dicot	SCA: 2733	–	14	13	182
	*Ipomea batatas* L	Dicot	–	Sweet potatoes	5	4	20
Cyperaceae	*Cyperus distans*	Monocot	–	–	63	62	3906
Dryopteridaceae	*Dryopteris felix-mas*(L.) *Schoot*	Dicot	–	Fern plant	9	8	72
Malvacea	*Urena lobata* L	Dicot	SCA: 7001	–	21	20	420
Maranthaceae	*Marantchloa purpurea* (Ridl.) *Milne-Redh*	Dicot	SCA: 1351	–	6	5	30
Nymphaeceae	*Nymphea alba* (H.)	Dicot	–	Water lily	8	7	56
Onagraceae	*Ludwigia abysisnica A. Rich*	Dicot	–	Water primerose	17	16	272
Poaceae	*Anzonopus compressus* (Sw.) *P. Beauv*	Monocot	–	Flat leaf carpet grass	5	4	20
	*Coix lachrymal-jobi* (L.)	Monocot	–	–	13	12	156
	*Cyndon dactylon* (Linn.)	Monocot	–	Bermuda grass	103	102	10,506
	*Echinochloa crus-pavonis* (Kunth) *Schult*	Monocot	SCA: 6075	–	20	19	380
	*Echinochloa paramidelis* (Lam.) *Hitchc*	Monocot	–	–	43	42	1806
	*Eragrostis* spp. *Michx*	Monocot	–	–	18	17	306
	*mperatea cylindica*	Monocot	–	Spear grass	10	9	90
	*Leersia hexandra Sw*	Monocot	–	–	68	67	4556
	*Panicum maximum Jacq. Var*	Monocot	–	Guinea grass	19	18	342
	*Paspalum conjugatum P. J. Bergius*	Monocot	–	Sour grass	21	20	420
	*Pennisetum purpereum* (Schumach)	Monocot	–	Elephant grass	21	20	420
Pteridiaceae	*Pteridium equilinum*	Dicot	–	Braken fern	7	6	42
Polygonaceae	*Polygonum limbatum Meisn*	Dicot	–	–	47	46	2162
Portuleraceae	*Portuleca oleraceae* L	Dicot	–	–	4	3	12
Rubiaceae	*Spermacocelatifolia Aubl*	Dicot	–	–	11	10	110
Sellaginellaceae	*Sellaginella Kraussiana* (Kunze)*A.Braun*	Dicot	–	–	2	1	2
Total	40				889		43,754

*SCA* = Southern Cameroon (code for the Limbe Botanic Gaderns), D = 0.055425, Simpson = 0.94.

**Table 5:** Simpson index of diversity from vegetation samples at Rural site (Mbelewa) in the Bamenda Municipality.

Family	*Species*	Class	Voucher no	Common name	n	n-1	n(n-1)
Acanthaceae	*Brilantasia nitens Lindau*	Dicot	–	–	22	21	462
	*Decliptera laxata C. B. Clarke*	Dicot	SCA: 1087	–	10	9	90
	*Justicia obliquifolia Standl*	Dicot	–	Blood medicine	5	4	20
	*Justicia flava* (Forssk.) *Vahl*	Dicot	–	Blood medicine	17	16	272
	*Nesonia canenscens Var. Smithii*	Dicot	–		7	6	42
Amaranthaceae	*Alternanthera sessalis* L	Dicot	–	Sessile joy weed	19	18	342
	*Amaranthus hybridus* L	Dicot	–	–	20	19	380
	*Amaranthuss spinosus* L	Dicot	–	Spiny amaranth	6	5	30
Apiaceae	*Centella asiatica* (L.) *Urb*	Dicot	–	Penny wort	5	4	20
Apocynacea	*Catharathus roseus*	Dicot	–	Periwinkle	20	19	380
Araceae	*Colocasia esculentus* L	Dicot	–	Cocoyam	4	3	12
Arecaceae	*Raffia fanivera Gaertnb*(L.)	Monocot	–	Raffia palm	27	26	702
Asteraceae	*Ageratum cornyzoides.* L	Dicot	–	King grass	24	23	552
	*Vernonia amygdalina Del*	Dicot	–	Bitter leaf	4	3	12
	*Vernonia gigantean (*Walter) *Trel*	Dicot	–	Wild large-leaf bitter leaf	2	1	2
	*Spilanthes filicaulis (*Schum. & Thonn.*) C. D. Adams*	Dicot	–	Eye for fowl	5	4	20
	*Synedrella nodiflora Cabi*	Dicot	–	Node weed	3	2	6
Athyraccea	*Diplazium zammati* (Khhn) *C. Chr*	Dicot	–	Single branch fern in fold	2	1	2
Commelinaceae	*Commelina beghalensis* L	Dicot	SCA: 1225	–	19	18	342
	*Commelina diffusa* (Burn) *F*	Dicot	–	Tropical sidewort	10	9	90
Convolvulaceae	*Ipomoea aquatic Forsk*	Dicot	SCA: 2733	–	17	16	272
	*Ipomea batatas* L	Dicot	–	Sweet potatoes	7	6	42
Cyperaceae	*Cyperus haspen*	Monocot	–	–	21	20	420
	*Cyperus distans*	Monocot	–	–	16	15	240
	*Mariscus alternifolius* (Sensus hepper)	Monocot	–	–	14	13	182
	*Rhynchospora corymbosa* (L.) *Britton*	Monocot	–	–	12	11	132
Dryopteridaceae	*Dryopteris felix-mas*(L.) *Schoot*	Dicot	–	Fern plant	22	21	462
Fabaceae	*Centrosoma Pubescens (Ridl.) Milne-Redh*	Dicot	SCA: 2526	–	19	18	342
	*Desmodium salicifolium (Poir.) DC*	Dicot	–	–	5	4	20
Malvacea	*Urena lobata* L	Dicot	SCA: 7001	–	20	19	380
Maranthaceae	*Marantchloa purpurea (Ridl.) Milne-Redh*	Dicot	SCA: 1351	–	6	5	30
Onagraceae	*Ludwigia abysisnica A. Rich*	Dicot	–	Water primerose	47	46	2162
Piperaceae	*Piper umberlatum* (L*.*)	Dicot	–	–	4	3	12
Poaceae	*Coix lachrymal-jobi* (L.)	Monocot	–	–	70	69	4830
	*Cyndon dactylon* (Linn.)	Monocot	–	Bermuda grass	9	8	72
	*Digitaria sanguinalis* (L.)	Monocot	–	Crab grass	16	15	240
	*Echinochloa paramidelis (*Lam.*) Hitchc*	Monocot	–	–	97	96	9312
	*Leersia hexandra* Sw	Monocot	–	–	63	62	3906
	*Paspalum conjugatum P. J. Bergius*	Monocot	–	Sour grass	12	11	132
	*Pennisetum purpereum* (Schumach)	Monocot	–	Elephant grass	3	2	6
	*Saciolepis Africana*	Monocot	SCA: 2835	–	20	19	380
Polygonaceae	*Rumex nepalensis* L	Dicot	SCA: 2540	–	8	7	56
	*Polygonium Limbatum Meisn*	Dicot	–	–	12	11	132
Pteridiaceae	*Pteridium equilinum*	Dicot	–	Braken fern	3	2	6
Rubiaceae	*Spermacoce latifolia Aubl*	Dicot	–	–	15	14	210
Sellaginellaceae	*Sellaginella Kraussiana (*Kunze*.)A.Braun*	Dicot	–	–	16	15	240
Smilalacaceae	*Smilax Krausiana Meisn*	Dicot	–	–	18	17	306
Solanaceae	*Physalis angulate* L. var	Dicot	–	Fejoo	2	1	2
Tilaceae	*Triumfetta cordifolia A. Rich*	Dicot	SCA: 5042	–	18	17	306
Zingiberaceae	*Aframomum officinalis Schum*	Monocot	–	–	10	9	90
Total	50				833		28,700

*SCA* = Southern Cameroon (code for the Limbe Botanic Gaderns), D = 0.041411, Simpson Index = 0.96.

### Variability of soil properties in the wetlands of Bamenda municipality and the relationship with macrophyte diversity

Table 6 shows the mean physicochemical properties of soils in the three major sites assessed. The soils were slightly acidic. The soils of the urban site (Mulang receiving drains from the urban zone) had a lower pH-H_2_O of 5.35 than pH-H_2_O of 6.04 of the rural site soils. The urban soils with the lowest pH also had the least diversity of plants. The urban soils also registered the highest EC (309 uS/cm) while the lowest EC value (120 uS/cm) was obtained at the Peri-urban site of Mile four. Soil organic carbon was in the order of 14.38% > 13.88% > 13.30% for the urban, rural and peri-urban sites, respectively. Organic carbon, however, showed moderate variation at the control and peri-urban site relative to its high variability at the urban site. Just like the soil organic matter, the soils of the urban zone had the best C/N ratio of 14.9. The sum of the exchangeable bases across the study sites was similar (0.97, 1.09, and 1.00 for the rural, peri-urban and urban sites, respectively). Exchangeable acidity stood in the order of 0.378 > 0.300 > 0.223 for the rural, peri-urban and urban sites, respectively. The concentration of exchangeable Al^3+^ was highest at the urban site.

**Table 6:** Physicochemical properties of soils within the Urban, Peri-urban and Rural wetland sites of Bamenda Municipality, Cameroon.

	Sand	Silt	Clay	pH (1:2.5)		EC uS/cm	OM	Tot N	C/N	Av. P mg/kg	Ca^2+^	Mg^2+^	K^+^	Na^+^	ΣBases	Exch. Acid	Al^3+^	H^+^	CECsoil	CECclay	ECEC	B.S. ECEC
Site	%			H_2_O	KCl		%				cmol( +)/kg					cmol( +)/kg			cmol( +)/kg			%
Rural site	57	23	20	6.04	5.41	230	13.88	0.55	16.8	18	0.30	0.50	0.04	0.1305	0.97	0.378	0.045	0.333	35.07	37.07	1.28	76.56
Peri-urban site	63	22	15	5.73	4.63	120	13.30	0.50	19.1	16	0.41	0.52	0.04	0.1265	1.09	0.300	0.170	0.130	35.01	58.25	1.19	91.60
Urban site	72	14	14	5.35	4.87	309	14.38	0.58	14.9	27	0.33	0.51	0.04	0.1239	1.00	0.223	0.193	0.030	39.37	71.36	1.15	86.96

These soils were evaluated for their variability using the coefficient of variation (CV %). CV values ranging from 0 to 15% are considered slightly variable, 15–35% moderately variable, while > 35% are considered highly variable^21,22^. From the data, (Table 7) Sand, pH-H_2_O, pH-KCl, and Na were consistently the least variable across the three sites. The sum of bases was moderately variable across the three zones. C/N, and Exch. Acidity values were highly variable.

**Table 7:** Variability classes of soil properties for the different sites in Bamenda municipality.

Site	Least variable (CV < 15%)	Moderately variable (15 < CV ≤ 35%	Highly variable CV > 35%)
Rural site	Sand, silt, clay pHH_2_O, pHKCl, EC, Na^+^, Al^3+^, CECsoil, ECEC	OM, Tot.N, Ca^2+^, Mg^2+^, K^+^, ΣBases, BSECEC	C/N, Av. P, Exch. acidity, H^+^, CECclay
Peri-urban site	Sand, pHH_2_O, pHKCl, K^+^, Na^+^, CECsoil, ECEC	Silt, clay, EC, OM, Av. P, Mg^2+^, ΣBases, BSECEC	Tot. N, C/N, Ca^2+^, Exch. Acidity, ECECclay
Urban site	Sand, pHH_2_O, pHKCl, Na^+^	Silt, Clay, Tot. N, Ca^2+^, Mg^2+^, K^+^, Na^+^, ΣBases, Al^3+^, CEC sol, B	EC, OM, C/N, Av. P, Exch. Acidity, H^+^, CECclay, BSCEC

### Clustering of the variables

**Figure 5:**
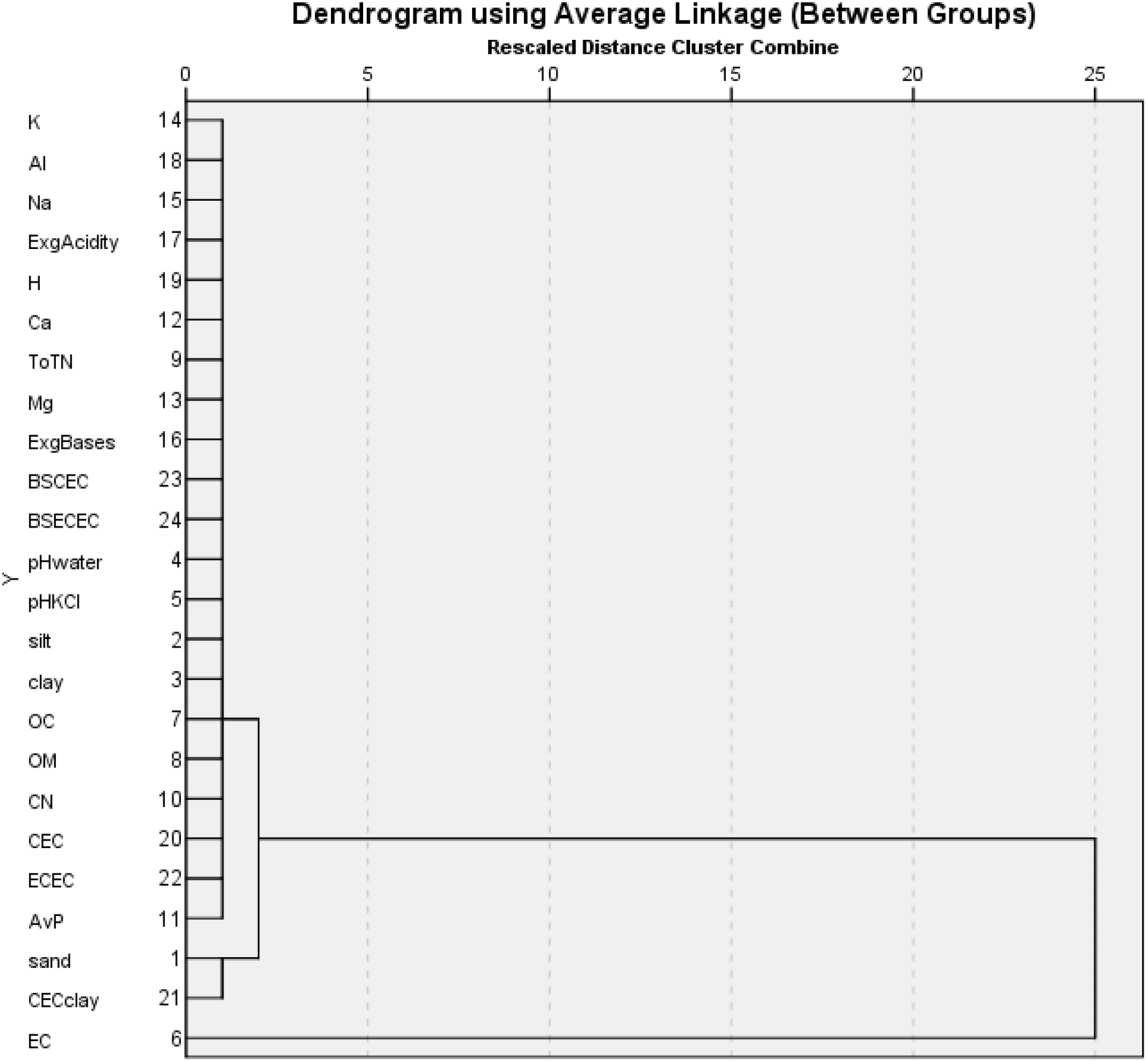
Dendrogram obtained from soil analyses in the freshwater wetlands of Bamenda Municipality.

Table 8 and Fig. 5 respectively show the agglomeration table and the hierarchical dendrograms for classification of the chemical variables for the surface soils from the wetlands. Two significant clusters were formed. Amongst the two major clusters, one cluster combined the rural environment (control site, site 21) and the peri-urban site where urbanization inputs are minimal. The other cluster represented the urban environment with varying human activities. In the urban zone. *Alternanthera sessilis* was widespread in this cluster. The Mann–Whitney U test revealed a significant (*P* < 0.05) lower exchange acidity in the rural than the urban site indicating contamination of the urban site while reducing its macrophyte diversity.

**Table 8:** Agglomeration schedule for different clusters obtained from soil analyses in the wetlands of Bamenda Municipality.

Stage	Cluster Combined		Coefficients	Stage Cluster First Appears		Next Stage
	Cluster 1	Cluster 2		Cluster 1	Cluster 2	
1	14	18	.002	0	0	4
2	23	24	.010	0	0	12
3	17	19	.015	0	0	5
4	14	15	.081	1	0	7
5	12	17	.338	0	3	7
6	9	13	.561	0	0	9
7	12	14	.840	5	4	9
8	20	22	1.192	0	0	19
9	9	12	1.681	6	7	11
10	4	5	1.820	0	0	12
11	9	16	7.061	9	0	14
12	4	23	30.756	10	2	14
13	2	3	112.000	0	0	17
14	4	9	129.863	12	11	18
15	7	8	399.432	0	0	16
16	7	10	849.348	15	0	17
17	2	7	908.425	13	16	18
18	2	4	2340.274	17	14	20
19	11	20	5437.587	0	8	20
20	2	11	9244.048	18	19	22
21	1	21	26,015.207	0	0	22
22	1	2	41,940.680	21	20	23
23	1	6	732,950.182	22	0	0

## Discussions

### Macrophyte species composition in the wetlands of Bamenda municipality

Macrophytes constituted an important part of the wetland. According to^23^, macrophytes (especially emergent and floating species) play a key role in the remediation of macro and micronutrients because they can grow vigorously in nutrient-rich environments. The plants could absorb and sequester pollutants, or reduce erosion by damping wave actions and as such should be considered for proper management to contribute significantly to the wellbeing of other components of the dynamic aquatic system.

Quadrat studies revealed 50 plants (macrophytes) species (mainly emergent herbaceous plants: grasses) distributed in 28 families.^24^ reported that grasses which are represented by 10.000 species and about 610 genera are the most widespread family of flowering plants in the world. Just like any aquatic ecosystem, these grasses were dominated by monocots. In some ponded areas close to the main river course, floating macrophytes were observed.^25^ did not identify such plants in the urban segment of the Mezam River system nor made mention of them in ponded areas proxy to it. The latter reported that in 2007, the Nkoup River system was characterized by eutrophic species (e.g. *Potamogeton spp.* and *Ceratophyllum demersum*) in the upstream segments that were considerably impacted by agricultural activities. The downstream and urban segments were dominated by the floating and emergent species, which accumulate and bio-concentrate significant amounts of metal pollutants. The fact that these plants were not reported in the agricultural wetlands in the Bamenda Municipality is an indication that this wetland is under stress activities and, thus continually degrading. Diverse urban amenities with some having considerably noxious activities have come into existence within the environs of this agricultural wetland of the municipality. These activities either impinge directly through physical alteration and development and/or indirectly through widespread diverse chemical inputs on the agricultural soils.^26^ commented on this alteration.

From the flora distribution, the rural site at Mbelewa showed the greatest level of species diversity (39 species). Here, the dominant species ranked in abundance were as follows *Raphia farinifera* > *Ludwigia hexandra* > *Coix spp.* > *Leersia hexandra* > *Ehchinochloa paramidelis.* Species biodiversity is used to indicate the ‘biological health’ of a particular habitat and is higher in less polluted areas. This indicates the rural site was less polluted.

The peri-urban site (Mile 4) which was represented by 40 species had the dominant species ranked in abundance as *Commelina bengalensis* > *Leersia hexandra* > *Cyperus distance* > *Ehchinochloa pyramidalis.* The dominance of *Commelina benghalensis* generally occurring in extensive uniform stands along the edge of the channel and in ponded and pond-like areas was similarly reported by^25^. These plants thrive well in seasonally flooded environments and as such their abundance and diversity are proof of their high tolerance for fluctuating water levels and anthropogenic disturbances in the area^23,27^.

Amongst the plants found in the urban site (at Mulang), the lining of the main river course with *Pennisetum purpureum*, *Echinochloa pyramidalis* and *Tithonia diversifolia* which have the same likelihood of occurring in wetlands as well as on dry land get these species into competition with *Coix sp., Poligonium* sp*., Ludwigia sp., Nesonia sp., Leersia sp., Echinochloa sp*, *Portulaca oleraceae* (obligate wetland species rarely occurring on dry land). This could thus lead to a reduction in diversity. *Portulaca oleracea* is a weed in cultivated fields and waste moist marshy places. It is also used as a vegetable and medicinal herb (^28,29^. Its presence in the area is therefore a high indication of environmental disturbance. *Penniseteum purpureum* among others is very effective in wastewater treatment^13^ and^25^ and therefore should be supported in the area of remediation. *Alternanthera sessilis* found in the area is a common species, widespread in waste and cultivated grounds, especially in damp or wet conditions^30^. It is an agricultural weed that invades disturbed wet areas in tropical and subtropical areas. *A. sessilis* has a low significant ecological impact^31^ and is thus a good indicator species. Just as, *A. sessilis, Rumex dentatus L.,* apart from its allelopathic activity (producing substances that inhibit or stimulate the growth of other plants near it), it also grows in disturbed habitats, often in moist areas. Their numerous nature in the urban wetland indicates a high level of environmental contamination.

### Simpson’s diversity indices of the area

Lower Simpson indices of diversity (0.94) recorded at the urban and peri-urban indicates that species diversity at the rural site (0.96) is slightly higher than that at the urban and peri-urban sites. The urban site had lower species abundances in certain areas, indicating worse environmental conditions such as toxic chemical inputs from the urban catchment. Similar findings due to worse.

environmental conditions have been reported by^4^ in a study to assess the diversity of macrophytes and the environment of the Ljubljanica River (Slovenia). The very close Simpson’s Diversity index value of 0.94 and 0.96 could be allied to the fact that in the study area there are no major industries that have heavy impacts on drain composition and thus soil and macrophyte diversity.^25^ have reported similar findings along River Mezam in the North West Region while^32^ have reported high variation in Simpson index of diversity for vegetation composition for mining-impacted sites in wetlands along Lake Victoria in East Africa. Though the difference between the rural and urban environment is slight, with continuous increase in urbanization, there is a future environmental concern, warranting monitoring.

### Variability of soil properties in the wetlands of Bamenda municipality and the relationship with macrophyte diversity

The lowest soil pH-H_2_O in the urban site at Mulang could be attributed to the fact that the site receives drains from the urban zone while peri-urban and rural do not. Soil pH plays a primordial role in nutrient availability to plants and therefore their growth, development and diversity. Low pH reduces the mineralization of soil organic matter and other nutrient reserves, inhibiting root growth and consequently, adsorption of nutrients. The acidic nature of the soil at the urban site can also be attributed to various anthropogenic factors. The soils of the urban wetland site (Mulang) site registered the highest EC of 309 uS/cm while the lowest EC value (120 uS/cm) was obtained at the Peri-urban site of Mile four. The highest EC which was recorded in the urban site could be associated with additional input ions from industrial urban activities. Soil organic matter is a good indicator of soil fertility and one of the most important soil components, along with stabilization of soil structure and improving infiltration rate. Nowadays, soil organic matter stabilization is perceived as a mechanism for organic carbon storage in the soil. Organic matter supplies energy and cell-building constituents for most microorganisms and is a critical factor in soil fertility. Organic carbon, however, showed moderate variation at the rural and peri-urban sites but was highly variable at the urban site (probably due to irregular inputs from anthropogenic activities in the urban centers including organic wastes from markets, and households) with a lower diversity of species. Just like the SOM, the soils of the urban zone had the best C/N ratio of 14.9. This means that the mineralisation rate of the SOM is good. The sum of the exchangeable bases across the study sites was similar (0.97, 1.09, 1.00 for the rural site, peri-urban site and urban site, respectively). Exchangeable acidity stood in the order of 0.378 > 0.300 > 0.223 for the control site, peri-urban and urban site respectively. However, the concentration of exchangeable Al^3+^ was highest at the urban site and a probable indication of inputs from industrial inputs from the urban site.

From the results, Sand, pH-H_2_O, pH-KCl, and Na were consistently the least variable across the three sites. ^33^ had similarly reported the least variability of soil pH for vertisols under rice cultivation in the Logone flood plain of Northern Cameroon. A very important parameter that influences many physicochemical properties of soils including the availability of nutrients, plant richness and diversity is soil pH. In this study, though the pH variability reported is small, minor changes in pH units have significant effects on nutrient availability. The moderately variable sum of bases was observed across the three zones of the study area which could be attributed to variation in levels of alluvial materials received. Also, variation in chrono-sequences of materials that have been subjected to different intensities of weathering could have a significant effect on these physical properties. All these factors have significant implications on nutrient availability to plants in the wetlands. The moderate variability of these bases implies that, for proper management of the wetlands, a unique policy for the area is insufficient to conserve the wetlands. The variability in C/N and exchange acidity could be attributed to organic materials, urban swept-off, and farm inputs that could have been deposited in these areas. The vegetation diversity across the zones could also be influenced by these parameters.

### Clustering of the variables

The two significant hierarchical dendrogram clusters that were formed correspond to the geographical location of the sampling sites and possible sources defining the soil quality like agricultural activity, industrial impact and fertilizing, which influences the diversity of macroflora in the area. Amongst the two major clusters, one cluster combined the rural environment and the peri-urban site where urbanization inputs are minimal. The other cluster represented the urban environment with varying human activities. In the urban zone. *Alternanthera sessilis* which was widespread in this cluster is a common species, very extensive in waste and cultivated grounds, especially in damp or wet conditions^30^. According to^34^, it impacts native plant species. The cluster on the lower part of the dendrogram represents dominantly rural and peri-urban areas with natural origins. This is supported by the dominance of *Commelina benghalensis,* which generally occurred in extensive uniform stands.

### Conclusion

The study concludes that effluents from drains to the urban, peri-urban and rural wetlands in Bamenda municipality, Cameroon are impacting the soils and plant species variedly. The diversity and variations in soil properties and plant species are influenced by human activities that are of different degrees in urban, peri-urban and rural areas. The variability between the soil properties in the different areas and the presence or absence of certain plant species indicates how to better manage the cluster to evade future problems. The chemical composition of soils in the urban cluster needs early remediation by encouraging the planting and monitoring of certain plants that can already take up the elements.

## Data Availability

The data sets used and analyzed during the current study are available from the corresponding author upon reasonable request.
